# β-Mannanase Production Using Coffee Industry Waste for Application in Soluble Coffee Processing

**DOI:** 10.3390/biom10020227

**Published:** 2020-02-04

**Authors:** Camila P. Favaro, Ilton J. Baraldi, Fernanda P. Casciatori, Cristiane S. Farinas

**Affiliations:** 1 Embrapa Instrumentation, Rua XV de Novembro 1452, São Carlos, SP 13560-970, Brazil; camilapfavaro@yahoo.com.br; 2 Graduate Program in Chemical Engineering, Federal University of São Carlos, São Carlos, SP 13565-905, Brazil; fernanda.casciatori@ufscar.br; 3 Department of Food Engineering, Federal University of Technology of Paraná, Medianeira, PR 85884-000, Brazil; baraldi@utfpr.edu.br

**Keywords:** bioprocess engineering, coffee carbohydrates, enzymatic hydrolysis, agro-industrial residues, solid-state fermentation (SSF), column bioreactor

## Abstract

Soluble coffee offers the combined benefits of high added value and practicality for its consumers. The hydrolysis of coffee polysaccharides by the biochemical route, using enzymes, is an eco-friendly and sustainable way to improve the quality of this product, while contributing to the implementation of industrial processes that have lower energy requirements and can reduce environmental impacts. This work describes the production of hydrolytic enzymes by solid-state fermentation (SSF), cultivating filamentous fungi on waste from the coffee industry, followed by their application in the hydrolysis of waste coffee polysaccharides from soluble coffee processing. Different substrate compositions were studied, an ideal microorganism was selected, and the fermentation conditions were optimized. Cultivations for enzymes production were carried out in flasks and in a packed-bed bioreactor. Higher enzyme yield was achieved in the bioreactor, due to better aeration of the substrate. The best β-mannanase production results were found for a substrate composed of a mixture of coffee waste and wheat bran (1:1 *w*/*w*), using *Aspergillus niger* F12. The enzymatic extract proved to be very stable for 24 h, at 50 °C, and was able to hydrolyze a considerable amount of the carbohydrates in the coffee. The addition of a commercial cellulase cocktail to the crude extract increased the hydrolysis yield by 56%. The production of β-mannanase by SSF and its application in the hydrolysis of coffee polysaccharides showed promise for improving soluble coffee processing, offering an attractive way to assist in closing the loops in the coffee industry and creating a circular economy.

## 1. Introduction

Coffee is widely produced and consumed globally. Brazil is the largest producer, followed by Vietnam and Colombia, with these three countries being responsible for more than half of the total supply of coffee. World coffee production in 2019/20 is projected to be 167 million bags, comprising 95 million bags of Arabica and 72 million bags of Robusta, while world coffee demand is projected to be 168 million bags. The use of instant coffee has increased, due to its advantages in terms of practicality, ease of preparation, and reduced wastage. Exports of soluble coffee have more than doubled over the last two decades, but remain small in volume terms, since over 90% of coffee is exported in the green form. Hence, most of the addition of value in the coffee industry occurs in the importing countries. In addition to market issues, many technical challenges still need to be overcome in order to expand the soluble coffee industry, especially in coffee producing countries [1].

For good soluble coffee productivity, a high yield is required in the extraction of the coffee polysaccharides, since water-soluble polysaccharides are the main components of the coffee extract. These polysaccharides are important for retaining volatile compounds and for providing the desired viscosity, ensuring satisfactory quality of the beverage [2]. The spent coffee grounds are rich in non-solubilized polysaccharides such as galactomannans, arabinogalactans, and cellulose, with half of the total being in the form of galactomannans [3,4,5]. Currently, thermal hydrolysis processes are used industrially to increase the extraction of polysaccharides, but these processes have high energy consumption and generate undesirable compounds that negatively impact the taste of the beverage [6]. A more eco-friendly and sustainable potential strategy for the hydrolysis and solubilization of coffee polysaccharides is to use an enzymatic route, since enzymes act under moderate process conditions and are highly specific. The main enzymes involved in degradation of the mannan structures are β-mannanase, β-mannosidase, β-glycosidase, and α-galactosidase [7]. Mannanases can be produced by a wide range of microorganisms, including different fungi [8,9,10,11] and bacteria [12,13,14].

Given the high costs of commercial enzymes, solid-state fermentation (SSF) can be an advantageous option that offers higher productivity of the enzymatic extracts, less susceptibility to inhibition, and greater stability of the enzymes towards variations of temperature and pH [15,16,17]. In SSF, microorganisms are cultivated on moist solid substrates, where the interparticle spaces are filled with air. Filamentous fungi adapt well to the low water activities of SSF systems, usually secreting enzymes into the extracellular medium during the growth phase. The benefits of using SSF for enzymes production are two-fold: the enzymes produced are of high economic value and they can be used in many different areas, including in the food industry [18].

The production of enzymes in SSF can be affected by culture conditions such as temperature, moisture content of the solid porous medium, pH of the solution impregnated into the particles, porosity of the substrate bed, aeration conditions, oxygen transfer, and the substrate type and composition. The residues generated in the coffee industry are potential substrates for the production of β-mannanases [19], since the enzymes produced using such a substrate should be more specific for degradation of this material. In addition, given the very large amounts of coffee residues produced worldwide, the reuse of this material could contribute to the development of industrial processes that have lower environmental and energy impacts. Recently, the production of cellulase enzymes by SSF using coffee husk as substrate has been demonstrated [20], thus showing the potential of this strategy. However, despite the wide range of biotechnological applications to valorize the spent coffee grounds residue [21,22,23], such as biogas and fertilizer production, its use as substrate for enzyme production still needs to be further investigated. 

Considering the above issues, this work evaluates the use of agro-industrial coffee waste to produce hydrolytic enzymes by SSF, focusing on the production of β-mannanase. Different fungi were tested, as well as combinations of the coffee waste with other substrates. The best operational conditions were defined at the flask scale, as well as using a column bioreactor. Fungal growth was correlated with enzymes production and the crude enzymatic extracts were applied in the hydrolysis of the coffee polysaccharides, in order to evaluate the ability of these enzymes to solubilize them, with the aim of improving soluble coffee processing, using an eco-friendly and sustainable strategy.

## 2. Materials and Methods 

### 2.1. Substrate

The medium roast Arabica coffee was obtained from Café Kühl (Limeira, São Paulo State, Brazil). The coffee residue was prepared by grinding the roasted coffee beans in a knife mill fitted with a 2–4 mm sieve. Extraction was performed with boiling water for 15 min, using a water:coffee ratio of 6:1 (v/w). The extract was filtered and the spent coffee was dried at 60 °C in an oven with air circulation, until reaching 3–5% moisture content. This residue was then sieved and separated into 1-2 mm particles for storage and subsequent use in the SSF and hydrolysis experiments. Wheat bran (obtained in São Carlos, São Paulo State, Brazil) and sugarcane bagasse (obtained in Piracicaba, São Paulo State, Brazil) were sieved, with particles >1 mm and 1–2 mm, respectively, being used in the SSF experiments.

### 2.2. Microorganisms

The fungal strains Aspergillus niger F12, Aspergillus niger 3T5B8, Aspergillus niger C, Aspergillus awamori 108(4), Aspergillus oryzae P6B8, Trichoderma reesei, and Trichoderma harzianum were used in this study. These fungal strains were obtained from the Embrapa Food Technology Collection (Rio de Janeiro, Brazil), except for the A. awamori 108(4) strain, which was provided by the Laboratory of Microbiology and Biomolecules (Center of Biological Sciences and Health, Federal University of São Carlos, São Carlos, Brazil). The stock culture was grown on a potato dextrose agar (PDA) medium, at 32 °C, until sporulation. A spore suspension was prepared in sterile Tween 80 (0.3%, *v*/*v*), for each slant culture. The spore concentrations in the suspensions were determined by counting in a Neubauer chamber.

### 2.3. Solid-State Fermentation in Flasks

A mixture of coffee residue and wheat bran (1:1, *w*/*w*) was first used as the solid substrate. The initial moisture content was adjusted to 50% (*v*/*w*) with Mandels medium [24], and the initial pH value was adjusted to 5.5. SSF was carried out in 250 mL Erlenmeyer flasks. After sterilization at 121 °C for 20 min in an autoclave, the culture medium was inoculated with 10^7^ spores per gram of substrate. The inoculated medium was incubated at 30 °C for 120 h. After the cultivation period, the enzymes were extracted by adding 50 mL of 0.05 M sodium citrate buffer (pH 5.3) to each 10 g of substrate and shaking (at 200 rpm) for 30 min at 30 °C. The liquid was filtered and subjected to centrifugation at 4 °C, 10,000 rpm, for 15 min. The clarified extract was stored at −18 °C prior to the analyses. Three biological replications were carried out for each test. The best fungal strain could then be selected from these experiments.

Following the same experimental procedure, using the chosen fungal strain, the composition of the substrate was varied, using coffee waste alone (CW), CW combined (1:1, *w*/*w*) with wheat bran (WB), and CW combined (1:1, *w*/*w*) with sugarcane bagasse (SB). Once CW combined with WB had been selected as the substrate, the CW:WB ratio was also varied, aiming to maximize β-mannanase activity. The results for substrate selection were subjected to Duncan′s multiple range tests, at a significance level of *p* < 0.05.

### 2.4. Solid-State Fermentation in a Column-Type Bioreactor

SSF cultivations were carried out in a column-type packed-bed bioreactor containing a previously inoculated medium, placed in a temperature-controlled water bath. The bioreactor system, consisting of so-called Raimbault columns, was composed of small glass columns (220 mm high, 24 mm outside diameter, and 20 mm internal diameter) within which the substrate was packed. Aeration of each column was achieved by percolating air (relative humidity of 80% at the inlet) through the bed of substrate, at a flow rate of 20 mL/min. The small quantity of medium packed within each column (a few grams) and the geometric characteristics of the glass columns make these bioreactors suitable for maintaining the ideal process temperature (since there is sufficient removal of heat through the wall) [25]. The cultivation conditions were 30 °C, 50% initial moisture content, and initial pH 5.0, with A. niger F12 growing in a mixture of coffee waste and wheat bran (1:1, *w*/*w*), for comparison with the SSF assays in flasks.

An addional set of cultivations in the column-type packed-bed bioreactor was carried out using a factorial design to evaluate the effects of temperature (X_1_), initial pH (X_2_), and initial substrate moisture content (X_3_) on the production yields of β-mannanase and β-glycosidase. The experimental design selected was a 2^3^ factorial design comprising eleven runs, corresponding to eight axial points and three central points, with the experiments carried out in random order. Each assay was performed in triplicate. The results of the factorial design assays were treated using STATISTICA 8.0 software (StatSoft, Inc., 2007). The selected conditions found in this factorial design was used to carry out the respirometric analyses by measuring CO_2_ in the outlet air stream leaving the columns of the bioreactor system, using a GMM 220 instrument (Vaisala, Vantaa, Finland). The cumulative amount of CO_2_ produced was calculated from the area under the CO_2_ vs. cultivation time curve.

### 2.5. Effect of Temperature and pH on β-Mannanase Activity

Cultivation experiments for the production of enzymes were performed in the column-type bioreactor at the selected conditions of 32 °C, initial pH 6.0, and addition of 8 mL of Mandels medium [24] to provide the desired moisture content for each 10 g of substrate composed of coffee waste and wheat bran (1:1, *w*/*w*). A central composite rotatable design (CCRD) was used to evaluate the effects on the enzymatic activity of two variables (temperature and pH) and their possible interaction. The experimental design selected was a central composite design comprising eleven runs, corresponding to four cube points, four axial points, and three central points, with the experiments carried out in random order. The dependent (response) variable was the β-mannanase activity. STATISTICA 8.0 software was used to analyze the experimental data, as well as to generate the ANOVA (analysis of variance) data and to plot the response surface. The assays were conducted using different pH values of the 50 mM sodium citrate buffer. The enzymatic activities were measured at different temperatures, adjusted using a temperature-controlled water bath. The substrate used was 0.5% (*w*/*v*) locust bean, prepared at the different buffer pHs, and the hydrolysis time was 20 min. A second order polynomial model of the form shown in Equation (1) was used to fit the data:

(1) $$Y={\;\beta }_{0}+{\;\beta }_{1}\cdot X_{1}+\beta_{2}\cdot X_{2}+\beta_{11}\cdot X_{1}^{2}+\beta_{22}\cdot X_{2}^{2}+\beta_{12}\cdot X_{1}\cdot X_{2}$$

where Y is the predicted response for β-mannanase activity, expressed as IU/g; β_0_ is the intercept term; β_1_ and β_2_ are the linear coefficients; β_11_ and β_22_ are the square coefficients; β_12_ is the interaction coefficient; and X_1_ and X_2_ are the coded independent variables (temperature and pH, respectively). The terms that were not statistically significant were removed from the model and added to the lack of fit. The thermostability of β-mannanase was evaluated during 24 h at 50, 60, 70, and 80 °C. 

### 2.6. Enzymatic Hydrolysis of Coffee Residue

The experiments were carried out in 5 mL tubes, using a loading of 5, 10, and 20% solids and 4 mL of crude enzyme extract. The enzymatic extract was produced by cultivation at 32 °C, with an initial pH of 6.0 and addition of 8 mL of Mandels medium to provide the desired moisture content for each 10 g of substrate composed of coffee waste and wheat bran (1:1, *w*/*w*). When using the commercial Cellic CTec3 enzyme cocktail (Novozymes, Araucária, PR, Brazil), a sufficient quantity of the powder was diluted in 4 mL of 0.05 M sodium citrate buffer (pH 5.3) to provide 2-5 FPU/g of coffee residue. Control of the hydrolysis pH was performed with the addition of 4 mL of 0.05 M sodium citrate buffer (pH 5.3). The supernatant was separated using two centrifugations, in Eppendorf tubes, for 10 min at 12,000 rpm and 4 °C. The progress of the hydrolysis was followed using the DNS method [26] to determine the reducing sugars released. The experiments and analyses were performed in triplicate.

### 2.7. Analytical Methods

The β-mannanase activity was determined by mixing 0.1 mL of an appropriately diluted enzyme sample with 0.9 mL of 0.5% (*w*/*v*) locust bean gum (Sigma G-0753) in 0.05 M sodium citrate buffer (pH 5.3), followed by heating at 50 °C for 20 min [27]. The reducing sugar released (as mannose) was determined by the dinitrosalicylic acid (DNS) method [26]. The activity was expressed in IU/g, corresponding to 1.0 μmol of reducing sugar released as mannose or glucose per minute and per g of solid that provided a given volume of enzymatic extract. The β-glucosidase activities were determined using a substrate of 150 mM cellobiose solution (Sigma, Saint Loius, MO, USA), prepared in 0.05 M sodium citrate buffer (pH 4.8) [28]. The reaction was carried out by incubating the enzyme:substrate (ratio of 1:1, *v*/*v*) solution for 30 min at 50 °C. The reaction was stopped by submersion in boiling water for 5 min. The glucose released was determined using a GOD-POD glucose test kit (Labtest, Lagoa Santa, MG, Brazil), following the procedure recommended by the manufacturer.

## 3. Results and Discussion

### 3.1. Effect of Substrate on Production of β-Mannanase

In the solid-state fermentations, evaluation was made of the use of coffee waste (CW) as the sole solid substrate, as well as in combination with wheat bran (WB) or sugarcane bagasse (SB), for the production of β-mannanase and β-glycosidase. The most appropriate solid substrate was considered to be the one that provided the highest production of these enzymes. Table 1 presents the enzymatic activities obtained for cultivation of *A. niger* F12 under SSF in flasks, at different times (72, 96, and 120 h), for all the solid substrates.

The use of a mixture of coffee waste and wheat bran as solid substrate during the SSF using *A. niger* F12 resulted in higher activities for both β-mannanase and β-glycosidase (Table 1). Wheat bran is considered a suitable waste for fermentation processes [29], with excellent industrial potential due to its high water retention capacity [30], good heat dissipation, and air circulation between the particles, allowing effective penetration of the fungal mycelium. It is also a complex substrate, acting as a source of carbon and nitrogen, in addition to being inexpensive [31,32]. These characteristics were observed in this work, since the wheat bran provided the fungus with an ideal environment for its growth, while the coffee waste served as an inducer for β-mannanase production, due to its high content of galactomannans.

**Figure 1:**
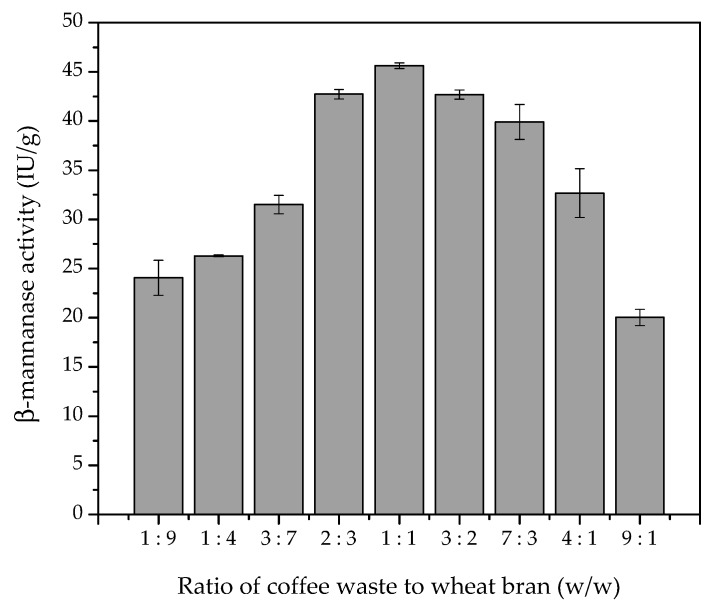
Effect of the ratio of coffee waste to wheat bran on the production of β-mannanase by *A. niger* F12 under SSF, at an initial moisture content of 50% (*v*/*w*), pH 5.0, and 30 °C.

In order to further evaluate the use of a combination of coffee waste and wheat bran as substrate for enzyme production, different ratios of these substrates were used during SSF (Figure 1). The ratio of coffee waste to wheat bran in the medium had a crucial effect on fungal growth and β-mannanase production. The data presented in Figure 1 show that the β-mannanase activity increased as the ratio of CW to WB increased, with the highest value reached at a ratio of 1:1 (*w*/*w*), followed by a decrease as the ratio was increased further. The statistical model identified the point of maximum enzymatic activity at a 1:1 ratio of coffee waste and wheat bran (R^2^ = 0.84), with small variations between ratios of 2:3 and 3:2 not affecting the enzyme production. Therefore, the 1:1 (*w*/*w*) ratio was chosen as the optimum for enzyme production in the subsequent experiments. These results indicate that the coffee residue is important to favor the production of β-mannanase, while the wheat bran acted as a nutrient source, providing the physical and chemical characteristics necessary for growth of the microorganism.

### 3.2. Effect of Different Fungi on Enzyme Production 

**Figure 2:**
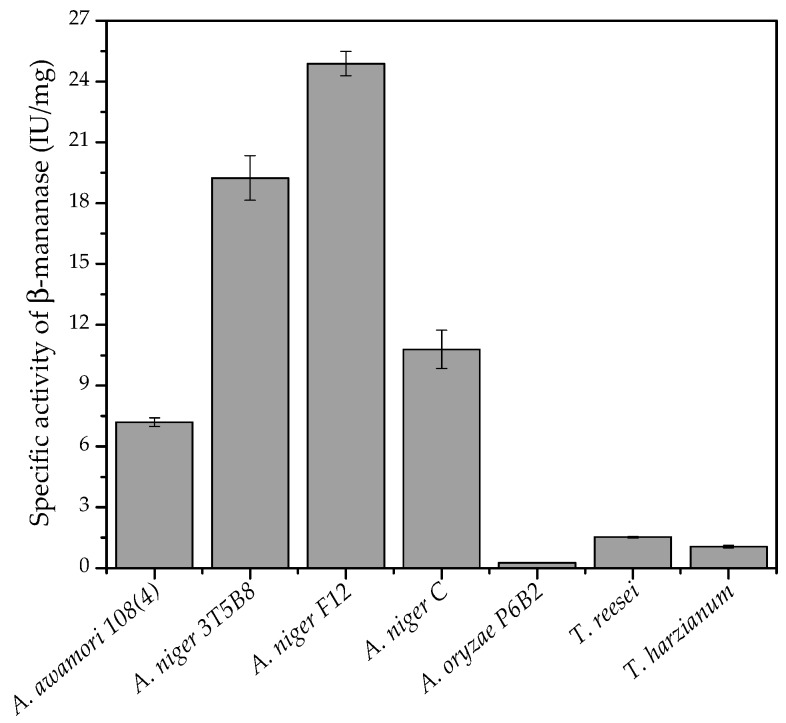
Specific activities of β-mannanase after SSF with different fungi for 120 h at 30 °C, pH 5.0, and 50% (*v*/*w*) moisture content.

Different filamentous fungi were studied for the production of β-mannanase under SSF, using the combination of coffee waste and wheat bran (1:1, *w*/*v*) as solid substrate. Figure 2 shows the specific activity of β-mannanase produced by each fungus, after 120 h of cultivation. The highest production of β-mannanase was obtained using *A. niger* F12, followed by *A. niger* 3T5B8. 

According to De Vries and Visser [33], strains of *Aspergillus* ssp. are the most important decomposers of hemicelluloses and celluloses in nature, having the capacity to produce several enzymes that degrade plant cell wall components. Ferreira and Filho [34] evaluated the production of β-mannanase by submerged fermentation using *T. harzianum* T4 and observed that the enzyme started to be produced on the sixth day of cultivation, reaching a peak on the eighth day, with activity of approximately 9 IU/mL. In the present work, β-mannanase production by *A. niger* F12 reached 8.6 IU/mL in five days, indicating better productivity of the enzyme under solid-state cultivation and with the use of this fungus.

### 3.3. Comparison of the Types of Cultivation Under SSF

The main advantage of SSF is the ability to use raw materials from agroindustry waste, but this technology has some drawbacks related to scale-up and the extraction of desired products in the downstream steps [35]. At the laboratory scale, flasks are usually used. In this case, the process occurs without forced aeration, is low cost, and is easy to handle, but it is difficult to control the operational parameters. The packed-bed bioreactor with glass columns (the Raimbault system) is provided with forced aeration through the static bed of particles, which helps to replenish O_2_ and water, while at the same time avoiding the accumulation of heat and CO_2_. In this work, both cultivation systems were evaluated. Table 2 shows the β-mannanase and β-glucosidase productivities obtained using flasks and the SSF column bioreactor. The β-mannanase activity on the third day of cultivation in the column bioreactor (52.6 ± 1.6 IU/g) was statistically equal to the activity achieved on the fifth day of cultivation in the Erlenmeyer flasks (51.9 ± 0.7 IU/g). The production of β-glucosidase was also higher using the column bioreactor. For both enzymes, cultivation in the bioreactor resulted in the highest productivity values after 72 h of cultivation. For β-mannanase, the productivity after 72 h was 50.7% higher when the bioreactor was used, rather than the flasks. Therefore, the results obtained for SSF in the bioreactor were promising, since there were higher production and productivity values for both enzymes, achieved in a shorter cultivation time, which would be favorable for improving the economic feasibility of the process.

Cerda et al. [20] used a solid-state fermentation bioreactor for the production of cellulase and xylanase, using coffee husks combined with other non-sterile residues as substrates. It was observed that the microorganisms acted synergistically in the production of the enzymes, with low gaseous emissions and reduced energy requirements making the bioprocess more environmentally friendly. García et al. [36] cultivated *Penicillium purpurogenum* in plastic bags, using coffee husks and coffee pulp as substrates, with the addition of sterilized cheese whey as a supplementary source of fermentable sugars (4.5–5.0% lactose), aiming at the extraction of total phenolic compounds. The coffee industry wastes supplemented with cheese whey were appropriate substrates for the growth of the microorganism and for obtaining phenolic compounds by the action of the enzymes produced during the process. In agreement with the earlier studies, the present findings showed that it was possible to create an environment that provided sufficient nutrients for both fungal growth and production of the desired enzymes. 

### 3.4. Optimization of β-Mannanase and β-Glucosidase Production in Bioreactor Fermentation

The important SSF variables analyzed for the optimization of enzymes production were temperature, initial pH, and initial moisture content. Table 3 presents the matrix of the assays, showing the real and coded values, together with the means and standard errors for the activities of β-mannanase and β-glucosidase (IU/g), determined in triplicate. The highest activities of the enzymes were found for the central point condition.

Considering the effects of the significant variables (99% confidence level), the largest and most significant effect was the curvature effect. This indicated that the production of these enzymes was optimized at the levels of the central point (tests 9, 10, and 11 in Table 3). 

Therefore, the experimental conditions of the central point were selected to produce the enzymatic extract to be characterized in terms of thermostability and the optimal conditions of pH and temperature for enzymatic activity. This extract was also used in the hydrolysis of the coffee polysaccharides. The optimum temperature for enzymes production by fermentation is usually close to the ideal temperature for growth of the microorganism, especially when the enzymes are products associated with growth, as in the case of the β-mannanase and β-glucosidase investigated here.

### 3.5. Time Profile of β-Mannanase Production

**Figure 3:**
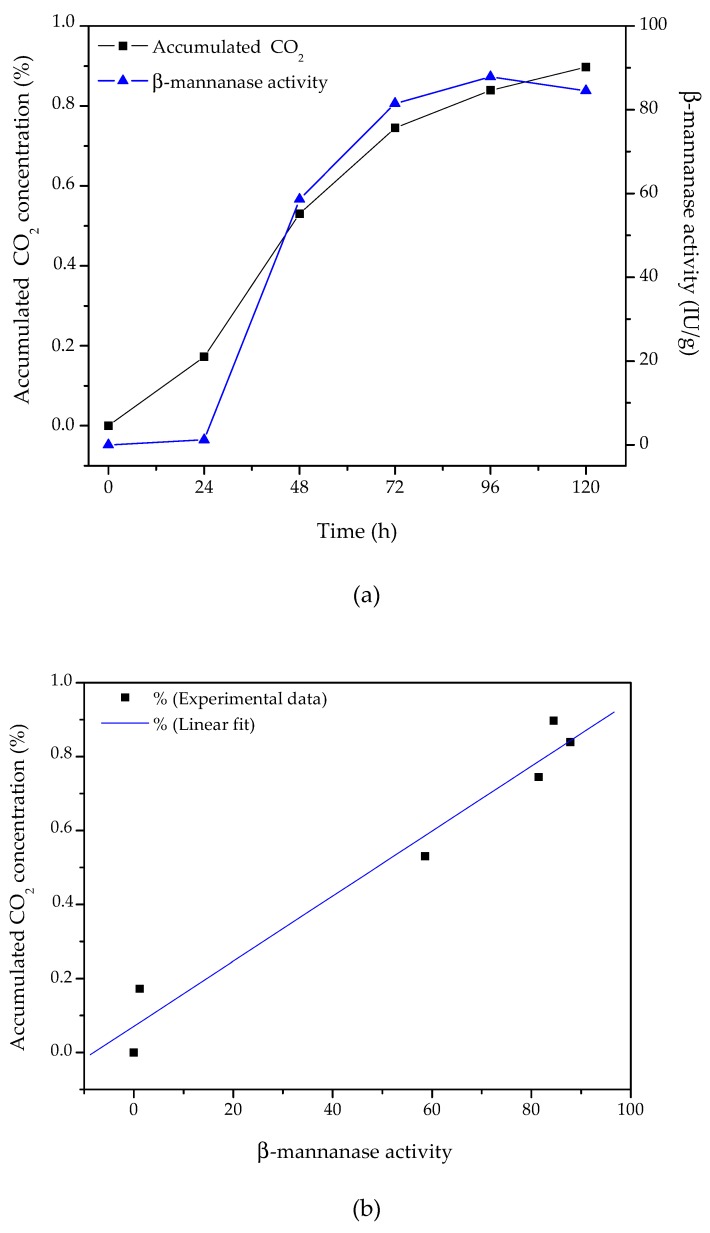

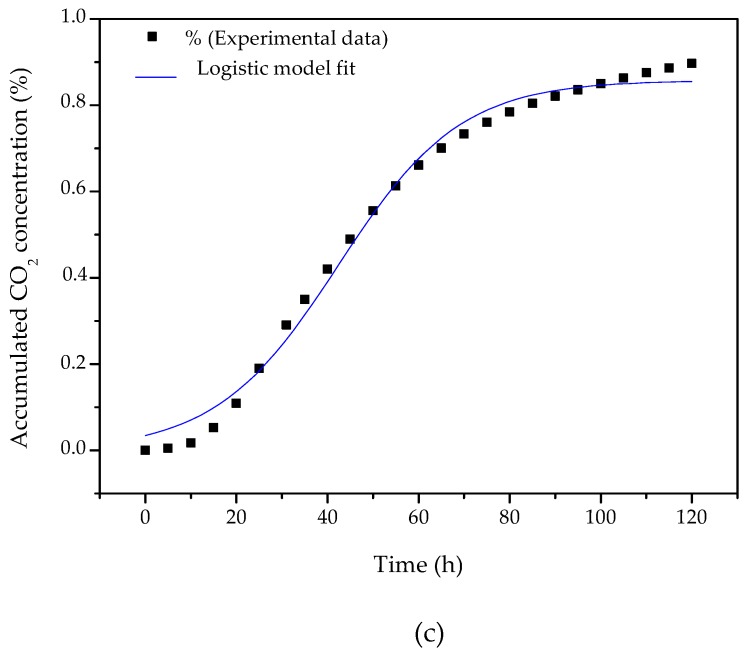
SSF for 120 h in the column bioreactor with *A. niger* F12 at 32 °C and pH 6.0. (**a**) Time-course curves for accumulated CO_2_ (left-hand y-axis) and β-mannanase activity (right-hand y-axis); (**b**) correlation curve; (**c**) logistic model fitting.

In SSF, the microbial biomass grows entangled in the solid substrate, which makes direct measurement of the microbial biomass concentration almost impossible. An indirect way to estimate fungal growth in SSF is by monitoring the behavior of respiratory gases (either the oxygen consumed or the CO_2_ produced by metabolism) [37]. A comparison of the β-mannanase produced and the CO_2_ accumulated during cultivation of *A. niger* F12 using an inlet air relative humidity of 80% and an air flow rate of 20 mL/min is presented in Figure 3.

The total amount of CO_2_ produced showed a good correlation with β-mannanase production (R^2^ = 0.963), especially after the first 24 h of cultivation. These results evidenced that the production of carbon dioxide during the cultivation period could provide valuable information about the progress of enzyme production.

According to Viccini et al. [38], who fitted kinetic models to a range of experimental results for microbial growth under SSF available in the literature, the logistic model provided a good fit to the results of most of the studies considered. Despite its mathematical simplicity, the logistic model can provide an adequate approximation of the complete growth curve, using a single equation, allowing visualization of the lag, fast growth, and stationary phases. The logistic model Equation (2) is an unstructured empirical model based on experimental observations:

(2) $$\frac{\mathrm{db}}{\mathrm{dt}}=\mu \cdot b\left(1-\;\frac{b}{b_{m}}\right)$$

where b is the fraction of microbial biomass in the fermented material, b_m_ is its maximum value, and µ is the specific growth rate. The initial condition adopted to solve Equation (2) was b = b_0_ (the fraction of biomass inoculated at the beginning of the process) at t = 0. The integrated form of the logistic model is given by:

(3) $$b=\frac{b_{m}}{1+\left(\frac{b_{m}}{b_{0}}-1\right)\cdot e^{-\mu t}}$$

When all conditions are optimal for growth, μ = μ_max_ (maximum specific growth rate). Fitting Equation (3) to the full curve of the experimental data for the accumulated CO_2_ concentration resulted in μ_max_ = 0.075 (± 0.004) h^−1^ (R² = 0.992, Figure 3c) for the bioprocess studied here. It should be noted that this parameter is specific for each combination of microorganism, substrate, and product, hence requiring specific experimental measurements of growth in the system of interest.

Mathematical modeling is a valuable tool for guiding the design, operation, and scale-up of SSF bioreactors, so the value of μ_max_ was used as an input for further simulation studies of the bioprocess studied here. Since metabolic heat generation is directly proportional to fungal growth, growth kinetics sub-models are commonly coupled with heat and mass transfer balance equations. The kinetics sub-models are usually empirical relations (in which μ_max_ appears as a parameter) that take account of the effects of parameters such as temperature and moisture content on microbial growth (expressed by the actual μ value) and, consequently, on the yields of growth-related enzymes [39,40,41,42,43].

### 3.6. Thermostability and Optimum Temperature and pH of β-Mannanase

**Figure 4:**
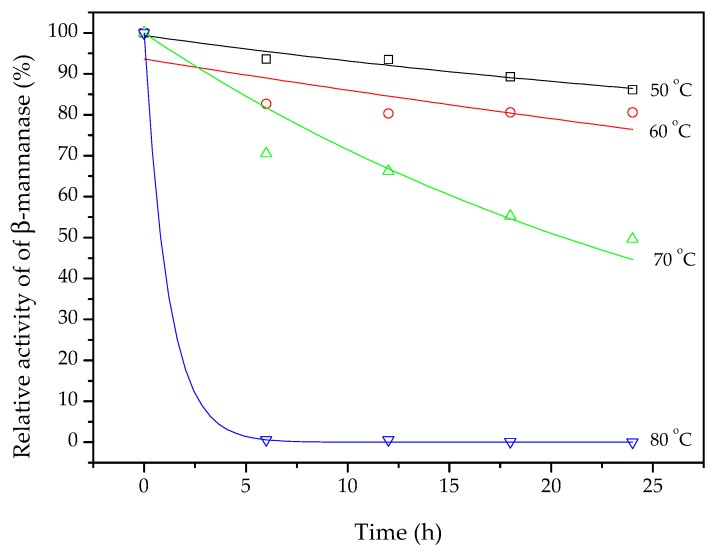
Thermostability of β-mannanase during 24 h at 50, 60, 70, and 80 °C. The symbols are the experimental data and the lines are exponential decay curve fits.

Thermostability can be defined as the activity retained after heating an enzyme extract at a selected temperature for a prolonged period of time [44]. After 24 h at 50 °C, the β-mannanase still had 87% of its original activity (Figure 4). After 12 h exposure at 50 °C, the enzyme retained 95% of its activity, while 80% was retained after 12 h at 60 °C. Soni et al. [45] produced β-mannanase using *A. terreus* FBCC 1369 under SSF, with coconut pulp flour as substrate, and evaluated the stability of the enzyme at 50, 60, 70, and 80 °C, during 60 h. After 24 h at 50 °C, the enzymatic extract had lost more than 10% of its activity, similar to the present findings. However, at temperatures from 60 to 80 °C, the extract lost 40% or more of its activity after 24 h. In the present work, around 80% of the β-mannanase activity was still retained after 24 h at 60 °C, showing that the enzyme produced under the conditions employed here was quite stable.

Since the amount of active enzyme can decline considerably following prolonged exposure to high temperatures, in many industrial applications the kinetics of enzyme deactivation can be as important as the kinetics of the reaction itself [46]. The simplest model of enzyme deactivation expresses the active enzyme concentration as a function of time, considering exponential decay (first order kinetics):

(4) $$E_{a}=E_{a0}\cdot e^{-k_{d}\cdot t}$$

where Ea is the active enzyme concentration, equal to Ea_0_ at time (t) zero, and k_d_ is the deactivation rate constant. According to Equation (4), the concentration of active enzyme (and consequently the enzyme activity) decreases exponentially with time.

The strong dependence of the rate of enzyme deactivation on the temperature can usually be satisfactorily described using the Arrhenius equation:

(5) $$k_{d}=A\cdot {e\;}^{\left(\frac{-E_{d}}{R_{g}\cdot T}\right)}$$

where A is the Arrhenius constant (or frequency factor), E_d_ is the activation energy for enzyme deactivation, R_g_ is the ideal gas constant (8.3145 J mol^−1^ K^−1^), and T is the absolute temperature. According to Equation (5), as T increases, the rate of enzyme deactivation increases exponentially.

The experimental data for the relative β-mannanase activity (Ea/Ea_0_, %) retained during 24 h at 50, 60, 70, and 80 °C were fitted using exponential decay curves (continuous lines in Figure 4). The model fit was only unsatisfactory for the data obtained at 60 °C (R² = 0.39). The values of k_d_ and R² obtained for the different temperatures are presented in Table 4. In addition, Equation (5) was fitted to the data of k_d_ as a function of temperature (in Kelvin). The Arrhenius model parameter values are shown in Table 4.

The deactivation energy for the β-mannanase was slightly lower, but of the same order of magnitude, compared to the usual range for many enzymes (170–400 kJ/mol) [47], so the Arrhenius model fitting confirmed the thermostability of the enzymatic extract produced in the present work. According to the kd values, a temperature increase of 10 °C, from 50 to 60 °C, doubled the rate of enzyme deactivation, while an increase from 70 to 80 °C increased the rate of deactivation 25-fold. Hence, it is clear that temperature has a critical effect on enzyme kinetics, so knowledge of the deactivation rate constant, as well as the Arrhenius model parameters, is essential in the design of successful applications for enzymes.

The effects of pH and temperature on the activity of the β-mannanase present in the enzyme complex produced by the filamentous fungus *A. niger* F12 grown on coffee waste and wheat bran, under SSF, were evaluated using statistical design of experiments and response surface methodology analyses. Table 5 presents the results of the full factorial design for β-mannanase activity under the different conditions of temperature and pH evaluated. Table 6 shows the coefficients of the mathematical model and the statistical parameters.

**Figure 5:**
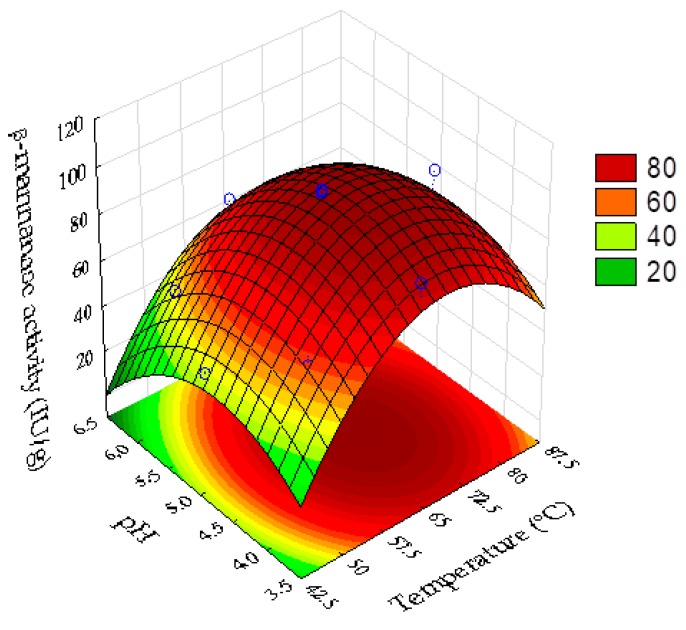
3D response surface plot for the activity of β-mannanase, considering the effects of the variables pH and temperature.

The analysis of variance (ANOVA) for β-mannanase showed a correlation coefficient (R²) of 0.925 and F_calc_ 4.06 times higher than the tabulated F value (95% confidence level). Hence, the statistical indexes were satisfactory for the prediction of the model used to describe the response surface plot of the enzyme activity as a function of pH and temperature (Figure 5). Equation (6) shows the quadratic model of the real β-mannanase activity as a function of pH and temperature:

(6) $$Y=\;-566.818\;+\;11.486\cdot X_{1}-0.08352\cdot X_{1}^{2}+114.05\cdot X_{2}-12.102\cdot X_{2}^{2}$$

where X_1_ and X_2_ are the uncoded values of temperature and pH, respectively. The non-significant term (synergistic effect between temperature and pH) was incorporated into the residuals for the ANOVA analysis.

The optimum temperature range for β-mannanase activity was between approximately 60 and 75 °C (Figure 5). The optimum pH range was between approximately 4.1 and 5.3. The best enzyme activity was at pH 4.8 and temperature of 69 °C. Under the optimal condition, the model predicted a β-mannanase activity of 96.7 UI/g. The model was successfully validated by experimental determination of the β-mannanase activity under the optimum point conditions, with a value of 96.4 ± 0.8 UI/g obtained.

### 3.7. Enzymatic Hydrolysis of Coffee Residue

In order to determine the best conditions for enzymatic hydrolysis using the crude extract produced by *A. niger* F12, solids loadings of 5, 10, and 20% were studied, aiming at maximizing the release of reducing sugars from the coffee waste. The results showed that the release of reducing sugars from the hydrolyzed solids increased linearly up to a loading of 20%. It is important to use high concentrations of solids, in order to obtain high concentrations of products. However, high solids concentrations can hinder agitation and mass transfer, leading to process losses. In this case, a 20% solids loading was chosen, since it provided a reducing sugars concentration of approximately 9 g/L when hydrolyzed with the crude extract (CE).

**Figure 6:**
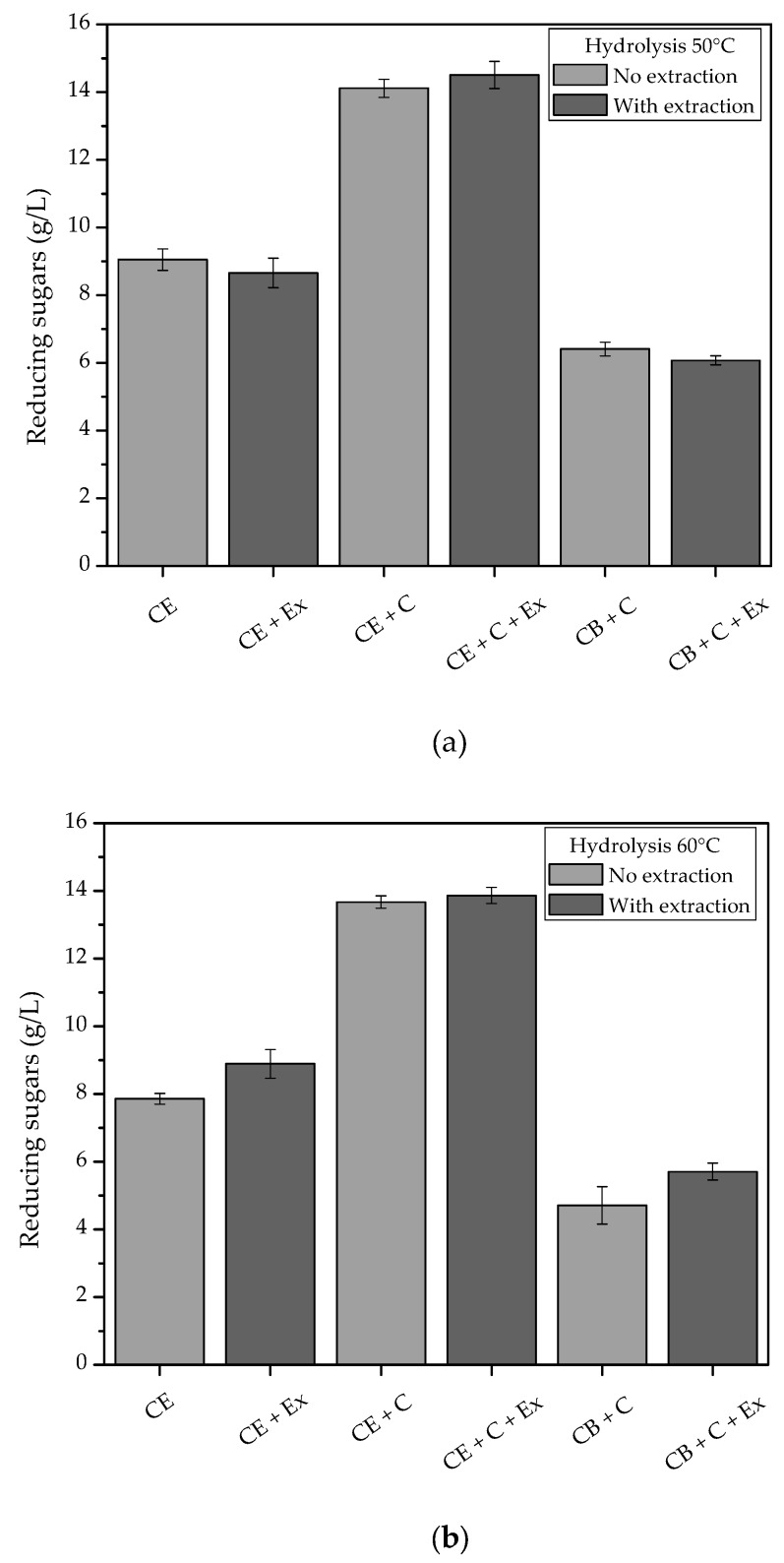
Concentrations of reducing sugars released from coffee residue using the crude enzyme extract produced here, with and without a commercial enzymatic cocktail, at (**a**) 50 °C and (**b**) 60 °C. Hydrolysis conditions: 30 rpm for 24 h at 20% (*w*/*v*) solids loading, with or without a thermal extraction period. CE: crude extract (enzymes produced in this work); Ex: 15 min extraction; C: Cellic CTec3 (5 FPU/g); CB: 50 mM sodium citrate buffer (pH 5.3).

The enzymatic extract produced by SSF was also used in combination with the commercial Cellic CTec3 enzymatic cocktail at 5 FPU/g, for the purpose of comparison. The combination of the crude extract with the commercial enzymes resulted in a 56% increase in hydrolysis, compared to the reaction using only the crude extract (Figure 6). The addition of an extraction step 15 min after the enzymatic hydrolysis did not significantly increase the release of reducing sugars. The results indicated the potential for further studies of enzyme production by SSF with coffee waste and the use of these enzymes in enzymatic hydrolysis applications in the soluble coffee industry.

Baraldi et al. [5] performed enzymatic hydrolysis with the fine extraction residue, using a commercial enzyme mixture for hydrolysis in a micro-reactor at 50 °C and pH 5.0. The time profile of reducing sugars release was very similar to that obtained here (data not shown). At 71 h of hydrolysis, Baraldi et al. [5] obtained approximately 9 g/L of reducing sugars in hydrolysis using a solids loading of 10%, with release of nearly 70% of the reducing sugars after around 25 h of reaction. In this work, 13.6 ± 0.4 g/L of reducing sugars was released after 72 h of hydrolysis, when a solids loading of 20% was used. After 24 h of reaction, the release of the reducing sugars reached around 70%, showing the potential of the enzyme cocktail produced in this study for hydrolyzing coffee polysaccharides, despite being in the form of an unpurified crude extract. 

In a recent study of the environmental impacts related to the recovery of cellulase produced in SSF with coffee husks [48], comprising evaluation of the fermentation, extraction, and purification stages of the process, it was observed that the main environmental impact was related to the high energy consumption in the downstream steps after fermentation, especially in the operations for enzyme purification. In the present work, the results showed that the enzymes produced by SSF using coffee waste as substrate were able to hydrolyze coffee polysaccharides, despite the fact that the enzymes were in the form of a crude extract. Although further studies should be performed concerning the concentration and isolation of enzymes, together with evaluation of their specificities, the above evidence suggests that the β-mannanase enzymatic extract produced here from soluble coffee processing waste may be promising for applications in the soluble coffee industry.

## 4. Conclusions

The findings of this work demonstrated the significance and potential of utilization of coffee residue, contributing to the development of innovative techniques to make better use of agricultural and industrial wastes. The production of β-mannanase was optimized using coffee waste and wheat bran (1:1, *w*/*w*) in the column bioreactor with cultivation of *A. niger* F12 under SSF. The coffee waste was important to favor the production of this enzyme, while the wheat bran was a good substrate to support the initial fungal growth. Under the optimal process conditions (considering temperature, pH, and moisture content), the crude enzymatic extract was highly effective in hydrolyzing coffee polysaccharides (14 g/L). Improvement of hydrolysis by the addition of a commercial cellulolytic enzyme cocktail highlighted the importance of the synergistic action of the enzymes. This work proposes a low-cost medium formulation that could be of industrial value, since β-mannanases have potential applications in the production of soluble coffee and in other food industries, as well as in animal feed, bio-bleaching of paper and pulps, and in the detergent industry, among others.

## Figures and Tables

**Table 1:** Enzymatic activities of β-mannanase and β-glycosidase obtained for SSF with *A. niger* F12 at 30 °C, pH 5.0, and 50% (*v*/*w*) moisture content, using different solid substrates.

Enzyme	Time (h)	Enzymatic Activity (IU/g) *		
		SB + CW	CW	WB + CW
β-mannanase	72	7.33 ± 0.44 c	10.16 ± 0.65 b	34.91 ± 0.75 a
	96	6.75 ± 0.84 c	15.42 ± 0.32 b	44.05 ± 0.85 a
	120	5.61 ± 0.59 c	15.94 ± 0.68 b	51.97 ± 0.67 a
β-glucosidase	72	45.36 ± 1.15 b	2.12 ± 0.08 c	67.54 ± 2.29 a
	96	51.80 ± 0.07 b	2.66 ± 0.26 c	69.85 ± 2.40 a
	120	47.93 ± 0.42 b	3.03 ± 0.05 c	72.54 ± 2.70 a

* Mean ± standard error; SB: sugarcane bagasse; CW: coffee waste; WB: wheat bran. Different letters in the same line indicate significant differences (Duncan′s test, *p* ≤ 0.05). The ratios of SB + CW and WB + CW were (1:1, *w*/*w*).

**Table 2:** Productivities of β-mannanase and β-glucosidase obtained in different types of cultures using *A. niger* F12 at 30 °C, pH 5.0, 50% moisture content, and 1:1 (*w*/*w*) coffee waste:wheat bran.

Time (h)	Productivity (IU/g.h) *			
	Erlenmeyer Flasks		Column Bioreactor	
	β-Mannanase	β-Glucosidase	β-Mannanase	β-Glucosidase
72	0.48 ± 0.01	0.94 ± 0.03	0.73 ± 0.02	1.00 ± 0.03
96	0.46 ± <0.01	0.73 ± 0.02	0.62 ± <0.01	0.82 ± 0.04
120	0.43 ± <0.01	0.60 ± 0.02	0.49 ± 0.05	0.66 ± 0.03

* Mean ± standard error.

**Table 3:** Factorial 2^3^ design for the production of enzymes (IU/g) in 72 h of SSF with *A. niger* F12 and 1:1 (*w*/*w*) coffee waste:wheat bran under different conditions of temperature, pH and initial moisture content.

Run	X_1_^a^	X_2_^b^	X_3_^c^	β-Mannanase (IU/g) *	β-Glucosidase (IU/g) *
1	−1 (29)	−1 (5)	−1 (5.6)	37.31 ± 0.46	35.58 ± 2.06
2	+1 (35)	−1 (5)	−1 (5.6)	39.26 ± 3.71	32.92 ± 0.91
3	−1 (29)	+1 (7)	−1 (5.6)	35.96 ± 0.47	40.01 ± 0.61
4	+1 (35)	+1 (7)	−1 (5.6)	44.71 ± 2.19	33.65 ± 2.12
5	−1 (29)	−1 (5)	+1 (10.4)	35.14 ± 1.03	29.87 ± 2.01
6	+1 (35)	−1 (5)	+1 (10.4)	42.62 ± 2.03	34.62 ± 0.98
7	−1 (29)	+1 (7)	+1 (10.4)	37.91 ± 2.00	36.29 ± 1.72
8	+1 (35)	+1 (7)	+1 (10.4)	49.65 ± 0.83	36.09 ± 1.67
9	0 (32)	0 (6)	0 (8)	59.03 ± 2.30	43.10 ± 1.60
10	0 (32)	0 (6)	0 (8)	62.40 ±2.30	43.62 ± 1.39
11	0 (32)	0 (6)	0 (8)	63.50 ± 2.97	41.77 ± 1.80

* Mean ± standard error; ^a^ X_1_: temperature (°C); ^b^ X_2_: pH; ^c^ X_3_: initial moisture content (the uncoded values within parentheses are the volumes of solution added to 10 g of solid substrate).

**Table 4:** Exponential decay and Arrhenius parameters for β-mannanase deactivation.

**Temperature (°C)**	**Exponential Decay**	
	**k_d_ (h^−1^)**	**R^2^**
50	0.006	0.93
60	0.012	0.39
70	0.033	0.90
80	0.833	0.99
	**Arrhenius Parameters**	
Frequency factor, A (h^−1^)	1.8 × 10^21^	
E_d_ (kJ mol^−1^)	147	
R^2^	0.85	

**Table 5:** Experimental conditions and results of the central composite rotatable design for the activity of β-mannanase produced by A. niger F12 in a column-type bioreactor at 32 °C and pH 6.0, with addition of 8 mL of Mandels medium.

Run	X_1_^a^	X_2_^b^	β-Mannanase Activity (UI/g) ^c^	β-Mannanase Predicted (UI/g) ^d^	Relative Error (%) ^e^
1	−1 (50)	−1 (4.0)	60.55	61.25	−1.15
2	1 (80)	−1 (4.0)	70.97	80.1	−12.87
3	−1 (50)	1 (6.0)	48.49	47.31	2.44
4	1 (80)	1 (6.0)	59.12	66.16	−11.9
5	−1.41 (43.8)	0 (5.0)	41.99	43.74	−4.17
6	1.41 (86.8)	0 (5.0)	80.38	70.38	12.44
7	0 (65)	−1.41 (3.59)	85.72	80.37	6.25
8	0 (65)	1.41 (6.41)	63.25	60.71	4.01
9	0 (65)	0 (5.0)	94.92	94.6	0.33
10	0 (65)	0 (5.0)	95.29	94.6	0.72
11	0 (65)	0 (5.0)	93.51	94.6	−1.17

^a^ Temperature (°C); ^b^ pH; ^c^ Experimental β-mannanase activity; ^d^ β-mannanase activity predicted by the model; ^e^ Relative error = ((X − $\hat{X}$)/X)*100, where X and $\hat{X}$ are the experimental and predicted values, respectively.

**Table 6:** Coefficient values (for coded variables) and statistical analysis for β-mannanase.

Source of Variation	Coefficient	*p*-Value
Mean	94.6	0.0000 *
Temperature (L ^a^)	9.43	0.0155 *
Temperature (Q ^b^)	−18.79	0.0018 *
pH (L)	−6.97	0.0446 *
pH (Q)	−12.1	0.0117 *
Temperature × pH	0.05	0.9891
R^2^	0.925	
F_calculated_	18.4	
F_calculated_/F_listed_	4.06	

* Significant at 0.05 level; ^a^ linear; ^b^ quadratic; F_4;6;0.05_ = 4.53.
